# JIP3 localises to exocytic vesicles and focal adhesions in the growth cones of differentiated PC12 cells

**DOI:** 10.1007/s11010-017-3222-7

**Published:** 2017-11-20

**Authors:** Patrick T. Caswell, Martin Dickens

**Affiliations:** 1 Wellcome Trust Centre for Cell-Matrix Research, Faculty of Life Sciences, University of Manchester, Manchester, M13 9PT UK; 2 Human Physiology, College of Health, Massey University, North Shore, Auckland, 0630 New Zealand

**Keywords:** JIP3, PC12 cells, Vesicles, JNK

## Abstract

The JNK-interacting protein 3 (JIP3) is a molecular scaffold, expressed predominantly in neurons, that serves to coordinate the activation of the c-Jun N-terminal kinase (JNK) by binding to JNK and the upstream kinases involved in its activation. The JNK pathway is involved in the regulation of many cellular processes including the control of cell survival, cell death and differentiation. JIP3 also associates with microtubule motor proteins such as kinesin and dynein and is likely an adapter protein involved in the tethering of vesicular cargoes to the motors involved in axonal transport in neurons. We have used immunofluorescence microscopy and biochemical fractionation to investigate the subcellular distribution of JIP3 in relation to JNK and to vesicular and organelle markers in rat pheochromocytoma cells (PC12) differentiating in response to nerve growth factor. In differentiated PC12 cells, JIP3 was seen to accumulate in growth cones at the tips of developing neurites where it co-localised with both JNK and the JNK substrate paxillin. Cellular fractionation of PC12 cells showed that JIP3 was associated with a subpopulation of vesicles in the microsomal fraction, distinct from synaptic vesicles, likely to be an anterograde-directed exocytic vesicle pool. In differentiated PC12 cells, JIP3 did not appear to associate with retrograde endosomal vesicles thought to be involved in signalling axonal injury. Together, these observations indicate that JIP3 may be involved in transporting vesicular cargoes to the growth cones of PC12 cells, possibly targeting JNK to its substrate paxillin, and thus facilitating neurite outgrowth.

## Introduction

The c-Jun N-terminal kinases (JNKs), a subset of the mitogen-activated protein kinase (MAPK) family, integrate numerous extracellular stimuli and help coordinate many cellular functions. These include immune responses, oncogenic transformation, apoptosis, cell survival, migration and differentiation [1, 2].

JNKs are activated when phosphorylated by the MAP kinase kinases (MKKs), MKK4 and MKK7 [3–6], which are in turn activated by a variety of upstream MKK kinases, in a three-tiered signalling pathway referred to as a MAPK module [1, 2, 7]. Signalling specificity in the JNK module is achieved, in part, by a family of scaffold proteins, the JNK-interacting proteins (JIPs), that organise JNK pathway components into specific signalling complexes and may target them to specific cellular locations [8–14].

The JIP1, 2 and 3 proteins are highly expressed in neurones where they are required for JNK activation and likely to be involved in the regulation of neuronal apoptosis and differentiation. JIP1 and JIP2 are structurally related, whilst JIP3/JSAP and JIP4/JLP form a separate group, with JIP4 being involved in p38 activation [8–10, 13, 15–18]. The JIP3 molecular scaffold contains a JNK binding domain, a leucine zipper and several coiled-coil domains. JIP3 binds to JNK and other components of the JNK module, including the MKKK, MLK3 and MKK7, in a manner that facilitates JNK signalling and activation [11, 12, 19].

In addition to the JNK module, JIP3 also binds regulators of vesicle transport. These include the microtubule motor proteins kinesin [20–24] and the dynein complex, [25–27] and Arf6 [28], suggesting that JIP3 is associated with vesicles and/or plays a role in their movement. The striking subcellular distribution of JIP3 in neurones, where it localises to the synaptic terminals in fully differentiated adult neurones and the growth cones of developing neurites in cellular models of neuronal differentiation, is likely explained by the anterograde transport of JIP3 to the plus end of microtubules by kinesin [12, 19, 21, 23, 29, 30]. JIP3 association with dynein is less well understood but may be required for retrograde vesicular transport and signalling after axonal injury or for clearance of organelles from the synapse/growth cone [25–27, 31]. JIP3 homologues have also been identified in *drosophila* (Syd), *Caenorhabditis elegans* (UNC-16) and zebrafish, and mutations at theses alleles cause defects in axonal transport [20, 25, 30]. In JIP3^−/−^ mice, defects in axonal transport block the differentiation of neurons forming the telencephalic commissure leading to death shortly after birth [29, 32, 33] and cultured hippocampal neurons lacking JIP3 show decreased axonal elongation and regeneration [24]. Together these observations suggest that one of the functions of JIP3 is to act as an adapter, tethering vesicular cargoes to kinesin and targeting them to growth cones, enabling neurite outgrowth and proper neuronal differentiation.

In our study we have used biochemical fractionation and immunofluorescence microscopy in PC12 cells, differentiating in response to nerve growth factor (NGF) to explore the subcellular distribution of endogenous JIP3 in relation to JNK and a variety of vesicular and organelle markers.

## Materials and methods

### Antibodies

A Glutathione S-transferase (GST) fusion of murine JIP3b (residues 1-273) was expressed in *Escherichia coli* BL21-DE3 using the expression vector pGEX-4T3 and purified by glutathione agarose affinity chromatography as described previously [34]. The GST tag was removed with thrombin and purified JIP3 (1-273) used as an immunogen in the preparation of a rabbit polyclonal anti-JIP3 antiserum by Cambridge Research Bioscience (Cambridge, UK). JIP3 antibodies were further purified from the serum by affinity chromatography on GST-JIP3b immobilised on Affi-Gel 10 (Biorad).

A mouse monoclonal antibody against α-tubulin (clone B-5-1-2) was purchased from Sigma. Mouse monoclonal antibodies raised against Synaptotagmin I cytoplasmic region (clone 41.1), Rab3a (clone 42.2) and Clathrin light chain (clone 57.4) were purchased from Synaptic Systems (Gottingen, Germany). Sheep polyclonal anti-TGN38 antibody was purchased from Serotec (Oxford, UK; cat. no. AHP499). Rabbit polyclonal anti-Synaptotagmin IV was a gift from Dr. Mitsunori Fukuda (Fukuda Initiative Research Unit, Riken, Saitama, Japan). Rabbit polyclonal anti-synaptophysin was a gift from Professor Ian Forsythe (Dept. of Cell Physiology and Pharmacology, University of Leicester). Mouse monoclonal anti-rSec6 was purchased from Calbiochem (San Diego, CA, USA; clone 9H5). Mouse monoclonal anti-dopamine β-hydroxylase was a gift from Dr Liz Seward (Department of Biomedical Science, University of Sheffield). Mouse monoclonal to JNK (cloneG151-666) was purchased from Pharmingen.

Horse-radish peroxidase- (HRP-) conjugated anti-rabbit and anti-mouse secondary antibodies were purchased from GE Healthcare Life Sciences (Amersham, UK). The HRP-conjugated anti-sheep secondary antibody was purchased from Zymed. Texas red-conjugated anti-mouse secondary antibody (from donkey) used in immunofluorescence microscopy was purchased from GE Healthcare Life Sciences. TRITC-conjugated anti-rabbit was a gift from Dr. Raj Patel, and FITC-conjugated anti-mouse was a gift from Dr. Andrew Fry (both Dept. of Biochemistry, University of Leicester). Alexa-488-conjugated anti-rabbit secondary antibody (from donkey) was from Molecular Probes (Eugene, OR, USA). For some immunofluorescence experiments, primary antibodies were directly labelled with Zenon fluorophores using a rabbit IgG labelling kit according to the manufacturer’s instructions (Molecular Probes, Eugene, OR, USA).

### Cell culture and treatments

PC12 cells were cultured in Dulbecco’s Modified Eagle Medium (DMEM) supplemented with 5% FBS, 5% HS, 2 mM l-glutamine and 100 units/ml of penicillin/streptomycin. Cells were maintained in collagen IV-coated T-75 flasks (Nunc) at 37 °C in the presence of 5% CO_2_. For fractionation experiments 1.5 × 10^6^ PC12 cells were plated onto 10 cm dishes and grown for 24 h prior to NGF treatment and fractionation. For microscopy, PC12 cells were plated at a density 1 × 10^5^ cells per 22 × 22 mm collagen-coated coverslip and grown overnight prior to transfection and/or NGF treatment. PC12 cells were differentiated by exposure to 50 ng/ml Nerve Growth Factor (NGF) for 72 h. in normal culture medium. HEK-293 human embryonic kidney cells were cultured in DMEM with 10% FBS, 2 mM l-glutamine and 100 units/ml of penicillin/streptomycin. Rat pheochromocytoma PC12 cells and HEK-293 cells were kind gifts from Dr. Liz. Seward (University of Sheffield) and Dr. Sally Prigent (University of Leicester), respectively.

### Constructs and transfections

GFP-JNK1, 2 and 3 expression vectors were constructed using the Gateway cloning system (Invitrogen). Full-length cDNAs for JNK1, JNK2 and JNK3 were cloned between the AttB sites of the Gateway donor vector pDONR201. cDNAs were then transferred by in vitro recombination via the LR reaction into the destination vector pDEST53. This generated plasmids allowing expression of N-terminally tagged GFP fusions to the three JNK isoforms under the control of the constitutive CMV promoter (GFP-JNK1, GFP-JNK2 and GFP-JNK3). GFP-EEA1 and GFP-paxillin-α were gifts from Dr. Jim Norman (Dept. of Biochemistry, University of Leicester).

A plasmid allowing expression of Flag epitope-tagged JIP3 (pCMV2-FLAG-JIP3) was constructed by recombination of a Gateway donor vector containing the full-length JIP3b cDNA (pDONR201-JIP3b) and the destination vector pCMV2-FLAG-DEST.

Plasmid vectors were maintained in *E. coli* DH5α and DNA for transfection prepared using the QIAprep Maxiprep Kit (Qiagen, Manchester, UK). PC12 and HEK-293 cells were transfected with plasmid constructs (2 μg) using Lipofectamine 2000 according to the manufacturer’s instructions (Invitrogen).

### Subcellular fractionation

For cell fractionation, PC12 cells were washed with ice-cold PBS, scraped into ice-cold fractionation buffer (0.32M sucrose, 10 mM Hepes pH 7.4, 1 mM PMSF, 2 mM benzamidine, 1 mM Na_3_VO_4_, 0.5 mM DTT, 2.5 mg/ml each of pepstatin, antipain and leupeptin, 10 mM β-glycerophosphate, 2 mM sodium pyrophosphate, 2 mM EDTA) and homogenised by 20 passes of a dounce homogenizer and 5 passages through a 27G needle. The homogenate (*H*) was fractionated by differential centrifugation in a procedure adapted from published protocols [35–37] to generate very low- (P1), low- (P2), medium- (P3) and high-speed (P4) pellets and a high-speed cytosolic fraction (C) as indicated in Fig. 3a. Each pellet was washed and re-centrifuged to prevent cross-contamination of subcellular fractions. Whole cell homogenate (100 µg) and an equivalent proportion of each of the fractions were subjected to SDS-PAGE and western blotting as indicated in the figures.

The P4 microsomal fraction was further fractionated by centrifugation on 10–40% iodixanol gradients generated in 12 ml Sorvall centrifuge tubes using OptiPrep (Axis Shield) and a gradient mixer. The P4 pellet was re-suspended in fractionation buffer and 0.5 ml (2 mg/ml) was loaded directly onto the gradient and centrifuged at 245,000×*g* at 4 °C for 16.5 h. Fractions (0.5 ml) were collected sequentially from the top of the gradient and proteins precipitated by the addition of 125 μl trichloroacetic acid (TCA) and incubation on ice for 1 h. Protein precipitates were pelleted by centrifugation at 20,000×*g* at 4 °C for 15 min. and washed once in 70% ethanol, once in 80% acetone before resuspension in SDS-PAGE sample buffer prior to SDS-PAGE and western blotting.

### Western blot analysis

Cell fractions were separated by SDS-PAGE on 6, 8 or 10% Tris–Tricine Biorad mini-gels [38]. Proteins were transferred to PVDF membranes (Immobilon P, Millipore) using a semi-dry transfer cell (Biorad) and membranes blocked overnight in 5% powdered milk or 3% BSA in tris-buffered saline containing 0.1% Tween-20 (TBST). Membranes were blotted with primary antibodies as indicated and visualised using HRP-coupled secondary antibodies and enhanced chemiluminescent detection (GE Healthcare Life Sciences).

### Immunofluorescence microscopy

Differentiated PC12 cells on collagen IV-coated coverslips (22 × 22 mm) were washed in ice-cold PBS and fixed with 4% paraformaldehyde (PFA). All further incubations were performed at 25 °C. Fixed cells were permeabilised with 1% Triton X-100 and coverslips blocked with 3% BSA in PBS/0.1% Tween-20 (PBST). Primary antibodies were diluted in 3% BSA/PBST and incubated with coverslips for 1 h. Secondary antibodies were incubated with coverslips in 3% BSA/PBST for 45 min. After antibody incubations, cell nuclei were stained with 0.1 μg/ml Hoechst 33,258 for 10 min. Washes between incubations were with PBST. Finally, coverslips were washed once briefly in dH_2_O before being mounted on slides in Mowiol/0.1% DABCO prior to imaging.

For imaging, Z-stacks (0.2 μm apart) were captured using a ×100, 1.4 NA objective on a Nikon TE300 inverted microscope and 5-neighbour volume deconvolution was performed using OpenLAB software. Extended focus images shown are representative of at least three independent experiments.

## Results

### Co-localisation of JIP3 with JNKs at the growth cone

**Fig. 1:**
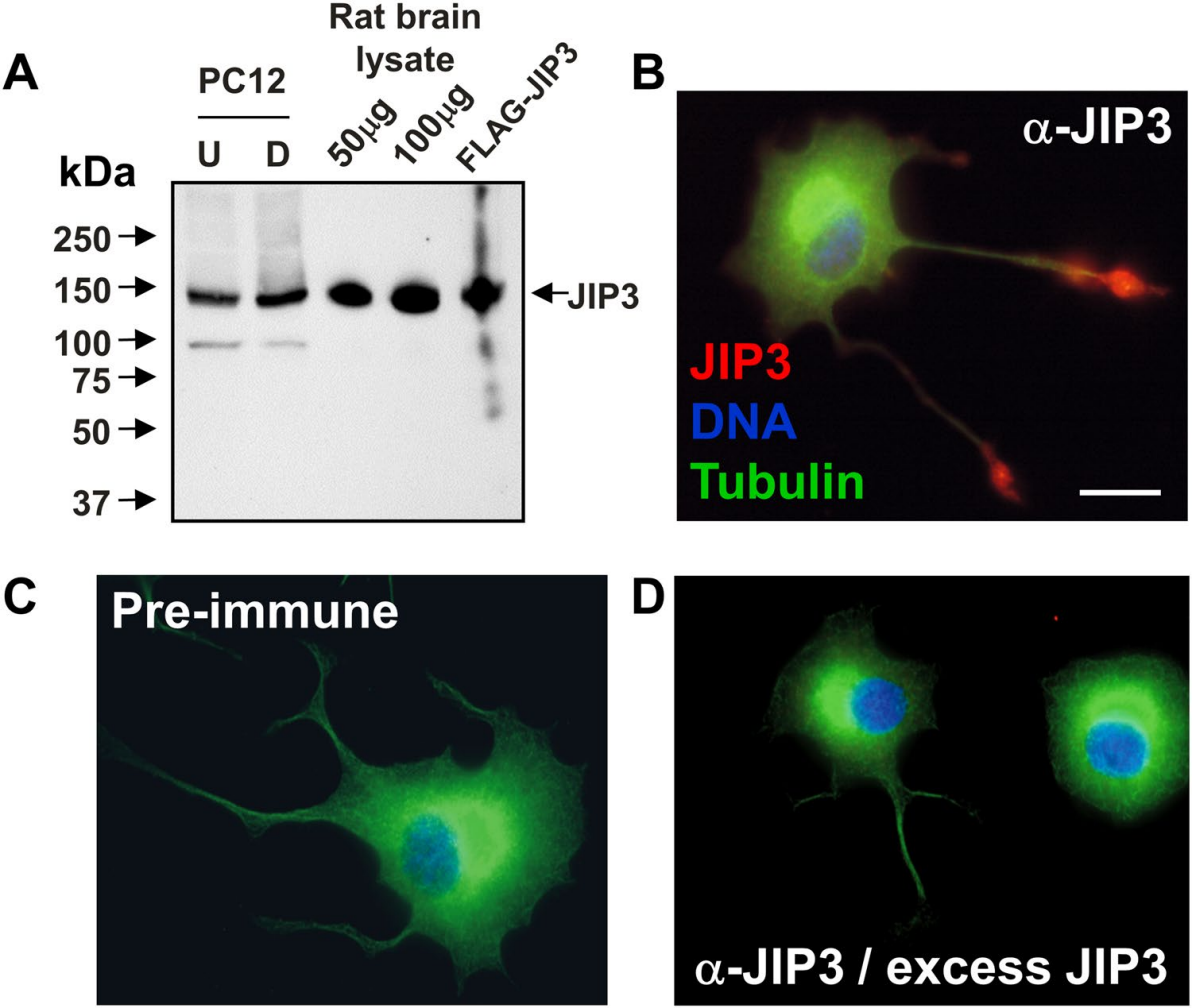
An affinity-purified JIP3 antiserum recognises endogenous JIP3 at the growth cone in differentiated PC12 cells. Lysates prepared from undifferentiated (*U*) or differentiated (*D*) PC12 cells (100 μg), rat brain (50 and 100 μg) and HEK-293 cells transfected with FLAG-JIP3b (100 μg), were separated by SDS-PAGE on 6% tricene gels. Proteins were transferred to blotting membranes and probed with affinity-purified JIP3 antiserum (1:1000) (**a**). PC12 cells were plated on collagen IV-coated coverslips, differentiated for 72 h, fixed in 4% paraformaldehyde and stained with antisera to α-tubulin (1:1000) and JIP3 (1:250) (**b**), pre-immune serum (**c**) or JIP3 antiserum in the presence of excess antigen (**d**). Primary antibodies were detected using anti-mouse FITC (1:200) and anti-rabbit TRITC (1:200) and nuclei stained with Hoechst 33,258. Images were captured using a Nikon TE-300 inverted microscope with a ×100, 1.4 NA objective and processed using Improvision OpenLAB software. Images shown are representative of three independent experiments. Scale bar 10 μm

In order to explore the subcellular distribution of endogenous JIP3, we generated and affinity-purified a rabbit polyclonal antibody against the N-terminus of JIP3b (residues 1–273). When used in western blotting, the purified antibody was able to detect endogenous, full-length JIP3 (150 kDa) with high specificity in lysates from PC12 cells, rat brain and also in HEK-293 cells transfected with a JIP3 expression construct (Fig. 1a). The JIP3 antibody also recognised endogenous JIP3 at the growth cones of differentiated PC12 cells as expected (Fig. 1b). Neither pre-immune serum, nor JIP3 antiserum incubated in the presence of excess JIP3 antigen, recognised JIP3 in PC12 cells, demonstrating the specificity of the antibody (Fig. 1c, d).

**Fig. 2:**
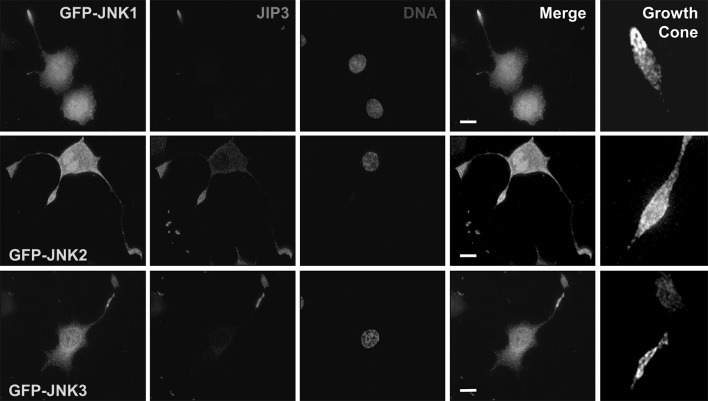
JIP3 co-localises with JNK isoforms at the growth cone in differentiated PC12 cells. PC12 cells, plated on collagen IV-coated coverslips, were transfected with constructs expressing GFP-JNK isoforms and differentiated for 72 h with NGF. Cells were fixed in 4% paraformaldehyde and stained with affinity-purified JIP3 antiserum (1:250) using a TRITC-conjugated anti-rabbit secondary antibody (1:200). Nuclei were stained with Hoechst 33,258. Images were captured using a Nikon TE-300 inverted microscope with a ×100, 1.4 NA objective and processed using Improvision OpenLAB software. Images shown are representative of three independent experiments. Scale bar 10 μm

JIP3 is a JNK pathway scaffold protein that interacts with JNK and upstream kinases. To test if JIP3 co-localised with JNK, we examined the distribution of endogenous JIP3 in differentiated PC12 cells expressing GFP-tagged JNK isoforms (Fig. 2).

All three JNK isoforms showed a diffuse, somewhat punctate distribution throughout the cell, being found in the cytoplasm, nucleus and in the growth cones at the tips of the neurites. In contrast, JIP3 was mainly concentrated at the growth cones (Fig. 2). In merged images, it was clear that all three JNK isoforms co-localised with JIP3 at the growth cone. This was particularly striking upon closer examination of iteratively deconvolved single-slice images of the growth cones, where there was significant co-localisation of JIP3 and JNK in distinct co-incident punctate structures that could represent vesicles, focal adhesion complexes or both.

### Subcellular fractionation of JIP3

**Fig. 3:**
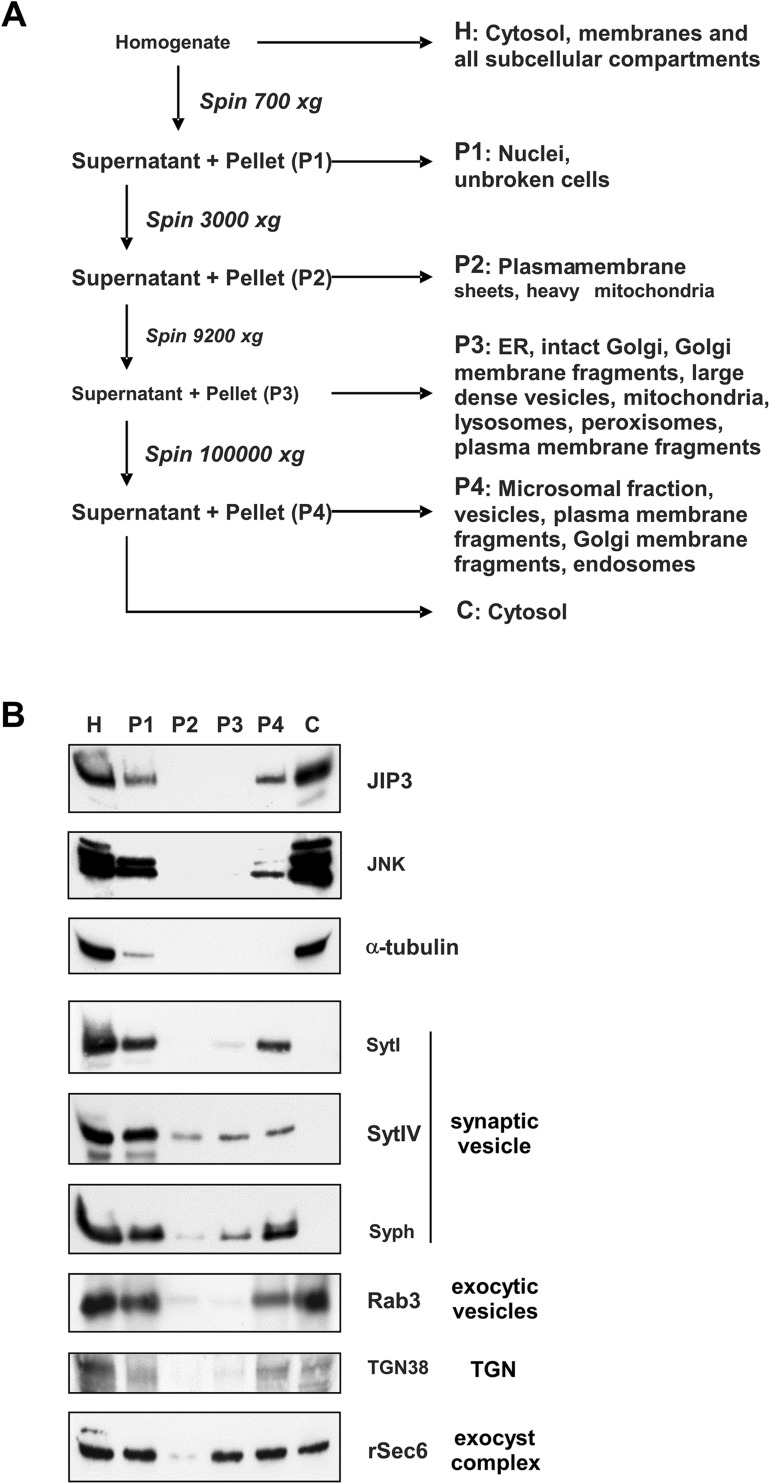

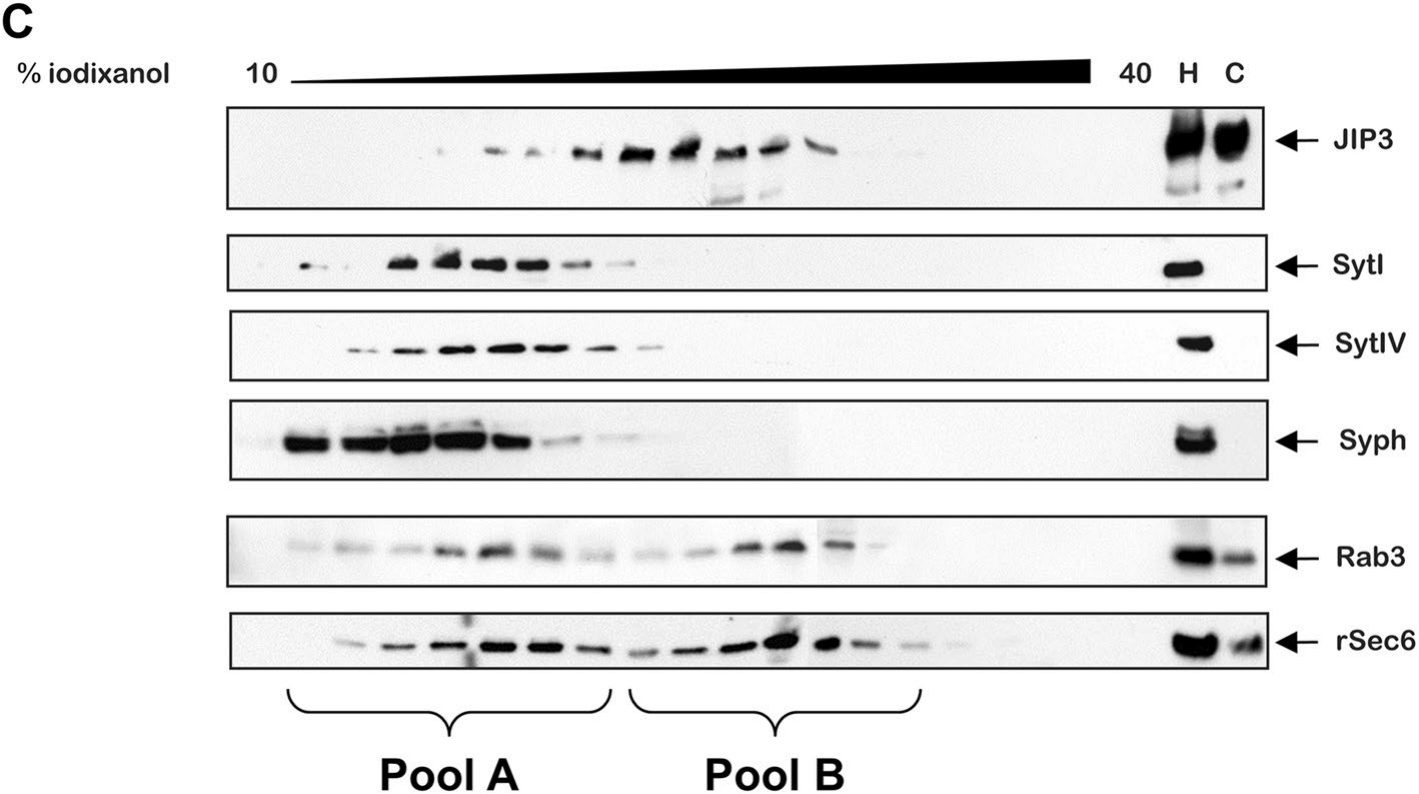
Subcellular fractionation of JIP3 differentiated PC12 cells. PC12 cells were differentiated for 72 h, homogenised and the lysate fractionated by differential centrifugation, separating membranous compartments from the cytosol, using the protocol shown (**a**). Fractions were subjected to SDS-PAGE on 6 or 10% tricene gels and proteins transferred to blotting membranes. Blots were probed with antibodies to JIP3 (1:1000), JNK (1:1000), α-tubulin (1:5000), synaptotagmin I (Syt I, 1:1000), synaptotagmin IV (Syt IV, 1:5000), synaptophysin (Syph, 1:1000), Rab3a (1:1000), TGN38 (1:500) and rSec6 (1:1000) (**b**). The microsomal fraction (P4 pellet) from differentiated PC12 cells was prepared using the crude subcellular fractionation protocol shown in panel A. The P4 pellet was re-suspended, layered on a 10–40% iodixanol gradient and centrifuged to further separate microsomal constituents. Fractions were collected and subjected to SDS-PAGE on 6% tricene gels and proteins transferred to blotting membranes. Blots were probed with antibodies to JIP3, synaptotagmin I and IV, synaptophysin, Rab3a and rSec6 as described in the previous figure (**c**). Results shown in the figure are representative of three independent experiments

JIP3 has been implicated in vesicular transport and appears to localise to punctate vesicle-like structures. However, the vesicular cargoes with which JIP3 is associated in PC12 cells are poorly characterised. To resolve this, we initially employed a crude subcellular fractionation protocol involving sequential centrifugation of a PC12 cell homogenate in sucrose buffer to isolate various membrane-bound subcellular compartments (Fig. 3a). To verify the fractionation procedure and identify possible cargoes co-fractionating with JIP3 and JNK, cell fractions were blotted for JIP3, JNK and a panel of antibodies specific for vesicles and other organelles (Fig. 3b).

Both JIP3 and JNK were consistently co-distributed, being found only in the P1 pellet (probably due to unbroken cells), the P4 microsomal fraction and the cytosolic fraction, but not the lower speed P2 and P3 fractions. Although the majority of JIP3 and JNK were found in the cytosol, a significant proportion of the total was present in the microsomal fraction (P4), which includes vesicles. α-tubulin was primarily associated with the cytosolic fraction, although a small proportion was consistently found in the P1 fraction, probably due to unbroken cells. As expected, there was no detectable association of tubulin with any of the other membrane fractions (P2, P3 or P4). The synaptic vesicle markers synaptotagmin (I and IV) and synaptophysin are transmembrane proteins and so, as expected, were not present in the cytosolic fraction. All three were most abundant in the P1 and P4 fractions, with some synaptotagmin IV and synaptophysin also present in the P2 and P3 fractions. Rab3, an exocytic vesicle marker, was found at low levels in all fractions, but was considerably enriched in the P4 microsomal and cytosolic fractions. TGN38 is associated with the trans-Golgi network and post-Golgi vesicles and fractionated very similarly to Rab3. rSec6 forms part of the mammalian exocyst complex, and plays an important role in vesicle targeting and fusion during exocytosis [39]. We observed significant amounts of rSec6 in the cytosol and all membrane associated fractions except P2. These crude subcellular fractionation experiments show significant JNK and JIP3 co-distribution, with both being found in the P4 fraction, which also includes the vesicle-associated protein Rab3. Neither JNK nor JIP3 were seen in earlier-sedimenting fractions, suggesting that they are not associated with plasma membrane/Golgi fragments in the microsomal fraction as these organelles/fragments appear in the P2 and P3 fractions. Rather, JNK and JIP3 are likely to be associated with small vesicular membrane compartments.

To investigate this more closely, the microsomal fraction (P4) from differentiated PC12 cells was fractionated further by density gradient centrifugation on iodixanol gradients (10–40%) and fractions blotted for JIP3 and various vesicle markers as shown in Fig. 3c. Western blotting of the fractions for vesicular markers revealed two distinct, broad pools of immunoreactivity, likely corresponding to two vesicle populations with different density. A lower density pool (Pool A) across fractions 2–8 (~ 17–26% iodixanol) and a higher density pool (Pool B) across fractions 9–15 (~ 29–37% iodixanol). This was most obvious in the blots for Rab3 and rSec6 which were present in both pool A and pool B. The synaptic vesicle markers synaptotagmin (I and IV) and synaptophysin were confined to the lower density Pool A. The majority of JIP3 immunoreactivity sedimented across the higher density fractions in pool B, along with some of the Rab3 and rSec6, with little or no reactivity in the lighter density pool A. This suggests that JIP3 is not predominantly associated with synaptic vesicles but with a more dense vesicular fraction, which may also contain rSec6 and Rab3. Unfortunately, the small amount of JNK immunoreactivity we detected in the P4 fraction (Fig. 3b) was diluted to such an extent over the iodixanol gradient that, even after several attempts, we were unable to detect it in any of the fractions.

### Subcellular localisation of JIP3 with respect to vesicular markers

**Fig. 4:**
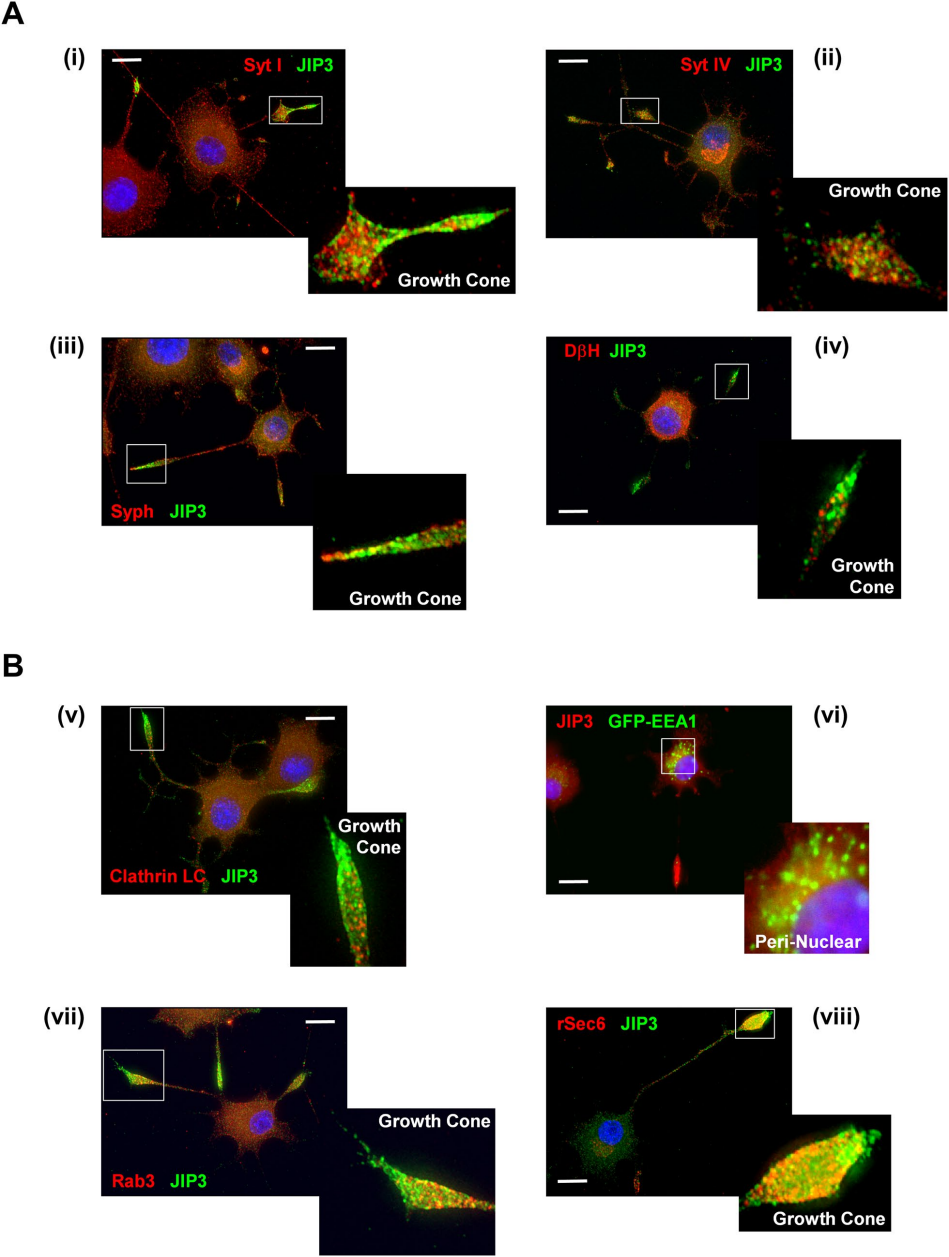
Localisation of JIP3 with respect to synaptic vesicle (**a**) and endo/exocytic pathway markers (**b**). PC12 cells, plated on collagen IV-coated coverslips, were differentiated for 72 h with NGF. Cells were fixed in 4% paraformaldehyde and stained with affinity-purified JIP3 antiserum (1:250). **a** Cells were counterstained with antibodies to the synaptic vesicle markers (i) synaptotagmin I (1:100), (ii) synaptotagmin IV (1:2000), (iii) synaptophysin (1:1000) or (iv) dopamine β-hydroxylase (DβH; 1:100). **b** Cells were counterstained with antibodies to the endo/exocytic vesicle markers (v) Clathrin LC (1:50), (vii) Rab3 (1:10), (viii) rSec6 (1:100). Prior to differentiation and staining for JIP3, some PC12 cells were transfected with a construct expressing GFP-EEA1 (vi). Primary antibodies were either directly labelled with Zenon fluorophores [Zenon-594 for synaptotagmin IV (ii) and synaptophysin (iii); Zenon-488 for JIP3 (iii)] or detected using fluorescently labelled secondary antibodies (Alexa-488 conjugated anti-Rabbit (1:10,000) or TRITC anti-rabbit (1:200) for JIP3; Texas Red anti-mouse (1:200) for SytI, DβH, Rab3, rSec6, Clathrin LC). Nuclei were stained with Hoechst 33,258. Images were captured using a Nikon TE-300 inverted microscope with a ×100, 1.4 NA objective and processed using Improvision OpenLAB software. Images shown are representative of three independent experiments. Scale bar 10 μm

To further explore the association of JIP3 with vesicles, we investigated the distribution of JIP3 with respect to vesicle markers using immunofluorescence microscopy. Differentiated PC12 cells were fixed and stained with affinity-purified JIP3 antiserum, Hoechst 33,258 and antibodies for synaptic vesicle markers (Fig. 4a) or exo/endocytic markers (Fig. 4b). As expected, the majority of JIP3 staining was concentrated at the growth cones of the neurites, with a distinct punctate distribution more obvious in higher-resolution deconvolved single-slice images (Fig. 4a, b).

The synaptic vesicle markers, synaptotagmin (I and IV), synaptophysin and dopamine c-hydroxylase all showed a similar punctate distribution throughout the cell body and the growth cones, with dopamine β-hydroxylase being less evident at the growth cones than the other markers (Fig. 4a, iv). Although the synaptic vesicle markers were co-located at the growth cone along with JIP3, the staining was rarely co-incident with the punctate JIP3 structures observed at higher magnification. This was entirely consistent with the behaviour of JIP3 containing vesicles on iodixanol gradient centrifugation, which appeared to sediment in a heavier fraction (Fig. 3c, Pool B) than those for synaptic vesicles (Pool A). Taken together these data strongly suggest that JIP3 is not associated with synaptic vesicles in differentiated PC12 cells.

Markers for the endocytic pathway, clathrin light chain (Clathrin LC) and the early endosomal antigen 1 (EEA1) showed little co-localisation with JIP3 in differentiated PC12 cells (Fig. 4b). Clathrin LC showed a diffuse staining pattern throughout the cell body with some staining evident at the growth cone that was not co-incident with JIP3 (Fig. 4b, v). EEA1 showed a punctate pattern of expression that was largely limited to the perinuclear region of the cell body with none being evident at the growth cone where the highest intensity of JIP3 staining was found (Fig. 4b, vi).

JIP3 however, did appear to show partial co-localisation with the exocytic markers Rab3 and rSec6 (Fig. 4b, vii, viii). Rab3 was found distributed throughout the cytoplasm and at the growth cone where there was some overlap with JIP3 staining. Although there was some staining of rSec6 evident in the cell body, the majority was observed at the growth cone where there was significant co-incidence with JIP3 staining. These observations are consistent with JIP3 partially co-sedimenting with Rab3 and rSec6 in pool B on iodixanol gradient centrifugation (Fig. 3c) and suggest that some JIP3 may associate with exocytic vesicles containing Rab3 and rSec6.

### JIP3 co-localises with paxillin at the tips of neurites

**Fig. 5:**
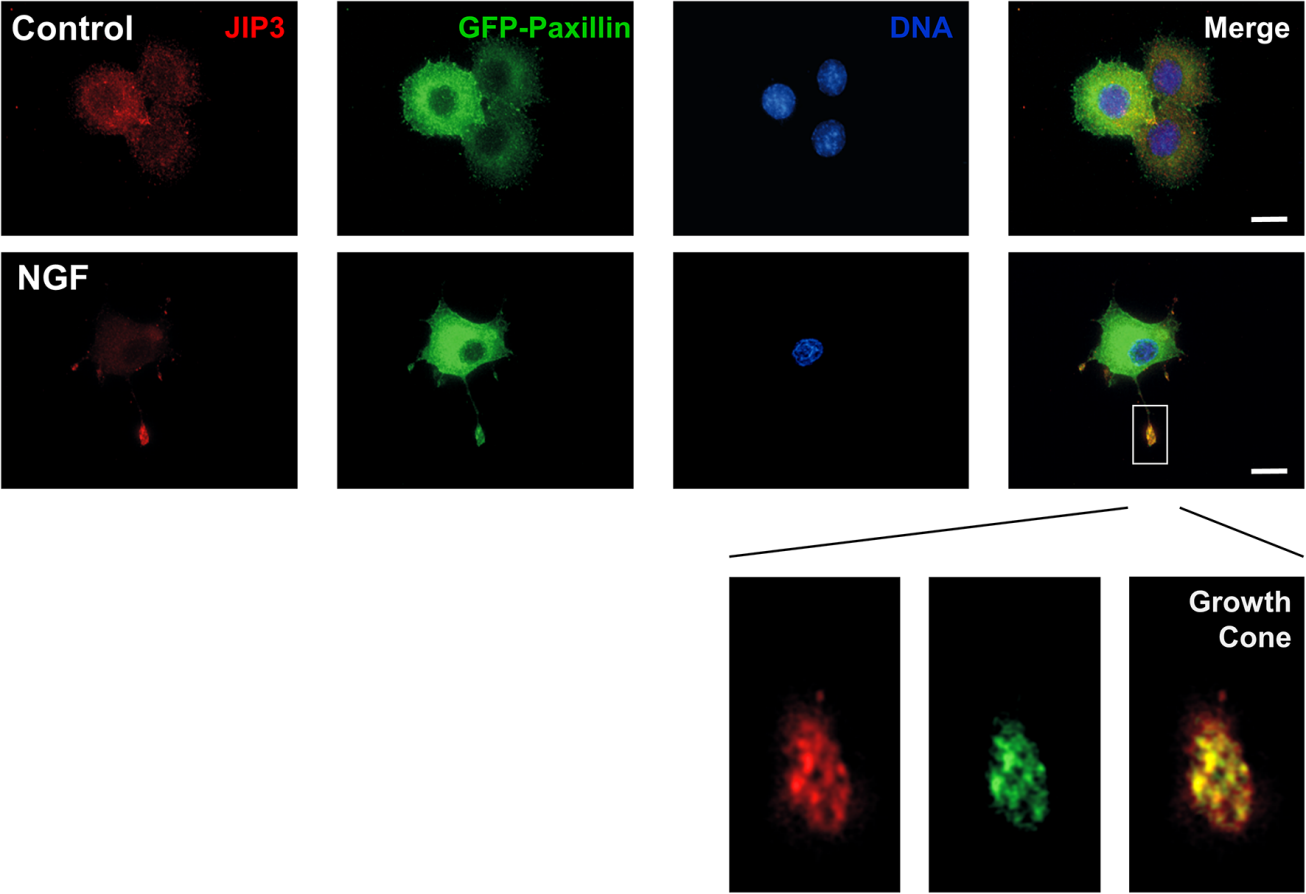
JIP3 co-localises with the JNK substrate paxillin at the growth cone in differentiated PC12 cells. PC12 cells, plated on collagen IV-coated coverslips, were transfected with constructs expressing GFP-Paxillin and differentiated for 72 h with NGF. Cells were fixed in 4% paraformaldehyde and stained with affinity-purified JIP3 antiserum (1:250) using a TRITC-conjugated anti-rabbit secondary antibody (1:200). Nuclei were stained with Hoechst 33,258. Images were captured using a Nikon TE-300 inverted microscope with a ×100, 1.4 NA objective and processed using Improvision OpenLAB software. Images shown are representative of three independent experiments. Scale bar 10 μm

Paxillin is a JNK substrate and component of focal adhesions that is implicated in cell migration and neurite outgrowth [40]. To test if JIP3 might be involved in locating JNK in focal adhesions at the growth cone, providing access to potential substrates involved in neurite outgrowth, we examined the distribution of paxillin with respect to JIP3 in PC12 cells expressing GFP-paxillin (Fig. 5). In undifferentiated PC12 cells, GFP-paxillin did not clearly co-localise with JIP3, but was found in the cell body, being excluded from the nucleus, and sometimes localised to focal adhesion-like structures at the cell periphery, which did not appear to contain JIP3 (Control). In differentiated PC12 cells, GFP-paxillin was again found mainly in the cell body and excluded from the nucleus. However, it was also found in the tips of developing neurites where paxillin was clearly co-located in structures at the growth cone that also contained JIP3 (NGF). These experiments identify a novel spatial association of JIP3 with the JNK substrate paxillin, suggesting that JIP3 might target JNK to the growth cone allowing it to phosphorylate paxillin and promote neurite outgrowth.

## Discussion

We have shown that the JNK pathway scaffold protein JIP3 co-localised with JNK isoforms at the growth cones of developing neurites in PC12 cells differentiating in response to NGF. Subcellular fractionation experiments and immunofluorescence microscopy showed that a significant fraction of the JIP3 in differentiated PC12 cells was associated with vesicular structures in the microsomal fraction. The JIP3-associated vesicles appeared to be distinct from endocytic vesicles and neurotransmitter containing synaptic vesicles, and more likely to be some type of exocytic vesicle involved in the development of the growth cone. There was a striking co-incidence of JIP3 and the JNK substrate paxillin at the growth cone, suggesting that JIP3 may be involved in targeting JNK to paxillin, allowing its phosphorylation, which may be required for extension of the growth cone.

### Localisation of JIP3 with respect to JNK

We developed an affinity-purified JIP3 antiserum to study the subcellular distribution of endogenous JIP3 in PC12 cells. The JIP3 distribution in differentiated PC12 cells was as expected, with striking localisation of JIP3 at the growth cones of neurites extending in response to NGF (Fig. 1). This distribution was highly consistent with the distribution of JIP family proteins seen previously in many neuronal cell types [12, 19, 21, 23, 29, 41]. In addition, JIP3 was found to co-localise with all three JNK isoforms at the growth cone in distinct co-incident punctate structures reminiscent of focal adhesions. This observation corroborates previous co-immunoprecipitation experiments indicating interactions between JIP3 and JNK isoforms [11, 12, 42–44].

Co-localisation of JNK with JIP3 at the growth cone suggests that JIP3 may be directly involved in JNK activation at the growth cone, perhaps in response to survival or guidance cues such as BDNF [22, 45]. It is also possible that JIP3 may deliver JNK-containing vesicles to the growth cone so that JNK can phosphorylate substrates important in axonal elongation and neurite outgrowth. JNK may also interact with JIP3 to regulate the transport of axonal vesicles, possibly by phosphorylation of motor or scaffold proteins, controlling the direction of transport, allowing switching between the +end-directed motor proteins kinesin and the −end-directed dynein [31, 46–49]. Retrograde transport of JNK from the growth cone mediated by JIP3-dependent association with dynein appears to be required for signalling back to the cell body in the response to nerve injury [27, 29, 50], or may be required for clearance of organelles from the synapse/growth cone [25–27, 31].

### Association of JIP3 with vesicles

To investigate the association of JIP3 with vesicles, we performed a biochemical subcellular fractionation of differentiated PC12 cells. Whilst the bulk of both JIP3 and JNK was found to be present in the soluble, cytoplasmic fraction, we also saw a significant proportion in the microsomal P4 fraction. Both JNK and JIP3 were undetectable in the P2 and P3 fractions, which contain larger organelles. This suggests that a fraction of the total JIP3 and JNK associate with vesicular structures but probably not large dense vesicles or larger organelles such as mitochondria, lysosomes or peroxisomes. Markers for exocytic pathway vesicles, including synaptic vesicles, were also found in the P4 fraction, along with JIP3 and JNK.

Density gradient centrifugation of the P4 microsomal fraction revealed two main pools of vesicles co-migrating with the exocytic markers Rab 3 and rSec6. A lighter pool (A) that also co-migrated with the synaptic vesicle markers Synaptotagmin (I and IV) and Synaptophysin, therefore is likely to be synaptic vesicles [51], and a denser pool (B) further down the gradient. In contrast, JIP3 fractionated in a broad but apparently single pool that did not significantly co-fractionate with pool A, showing that JIP3 is not likely to be present on synaptic vesicles in PC12 cells. There was significant co-fractionation of JIP3 and the denser pool B, suggesting that some of the JIP3 containing vesicles may be exocytic vesicles containing Rab3 and rSec6. JIP3 containing vesicles have previously been purified from synaptosomal fractions of whole mouse cortex using JIP3 antibody coupled to magnetic beads [52]. Subsequent analysis by electron microscopy and mass spectroscopy revealed two types of vesicles that were functionally and morphologically distinct: a large endocytic JIP3 vesicle, probably involved in the retrograde transport of signals to the cell body upon neuronal injury, and a smaller vesicle, likely transported in an anterograde direction and thought to be involved in axonal outgrowth and guidance [27, 31, 52]. The small vesicles identified did not contain significant quantities of synaptotagmin or synaptophysin, suggesting that they were not synaptic vesicles [51]. In contrast to these findings we observed what seemed to be a single pool of JIP3 containing vesicles in differentiated PC12 cells, but these were also negative for synaptotagmin or synaptophysin and therefore also unlikely to be synaptic vesicles [51]. Synaptosome fractions from whole mouse cortex likely contain vesicles from all of the cell types present in the cortex and it is possible that different neuronal cell types may have different populations of JIP3 vesicles according to their function. It is worth noting that the large JIP3 vesicles purified from mouse synaptosomes have much lower JIP3 staining than the smaller vesicles, suggesting that they are less abundant or contain less JIP3 [52]. It is possible that these larger retrograde JIP3 vesicles reported are absent from PC12 cells. The JIP3 vesicles we observe in PC12 cells appear to be most similar to the smaller anterograde vesicles thought to be involved in axonal growth [27, 32, 52, 53]. Vesicles such as these are likely to be more abundant in differentiating PC12 cells since they are extending neurites in response to NGF.

### Localisation with respect to vesicle markers

Immunofluorescence microscopy of differentiated PC12 cells corroborated our findings from cell fractionation. Imaging of JIP3 at the PC12 growth cone showed poor co-localisation with synaptotagmins (I and IV), synaptophysin and dopamine β-hydroxylase (Fig. 4a), confirming that JIP3 is unlikely to be associated with synaptic vesicles [51]. We also saw little evidence of JIP3 co-localisation with markers of the endocytic pathway including clathrin light chain and EEA1. This is in agreement with our fractionation data and supports the idea that differentiated PC12 cells do not contain significant quantities of the large retrograde JIP3 vesicles seen in cortical synaptosome preparations [31, 52]. JIP3 showed partial co-localisation with the exocytic vesicle markers Rab3 and rSec6 at the growth cone [39]. This is also in agreement with our fractionation data which showed both of these markers on pool A and pool B vesicles. Together, these findings suggest that JIP3 might be associated with some type of anterograde exocytic vesicle distinct from synaptic vesicles.

### JIP3 as a potential connection between vesicles and focal adhesions

The punctate staining of JIP3 at the growth cones, and co-localisation with vesicle markers and paxillin, suggests that JIP3 could be delivered to focal adhesions at the growth cone by vesicles. JIP3 acts as a downstream effector of Arf6 [28], a small GTPase that controls trafficking of integrin-containing vesicles and to regulate axonal outgrowth [54], suggesting that JIP3 could link vesicle trafficking to focal adhesion formation in developing growth cones. JIP3 has also been shown to interact with the focal adhesion kinase (FAK) and promote cell spreading [44, 55, 56], and JNK has also been found to localise to focal adhesions [57]. Together these observations suggest that JIP3 may influence aspects of cytoskeletal reorganisation, such as focal adhesion turnover, involved in the regulation of neurite outgrowth. Paxillin is a JNK and p38-MAPK substrate, associated with focal adhesions, involved in the regulation of cell spreading and implicated in cell migration and neurite outgrowth [40, 58–60]. Imaging of growth cones in differentiated PC12 cells expressing GFP-tagged paxillin showed a striking co-localisation of paxillin with JIP3, suggesting that JIP3 is a component of focal adhesions at the growth cone. JIP3 may therefore target JNK to paxillin, allowing its phosphorylation and the promotion of cell adhesion/spreading required for neurite outgrowth.

## Conclusion

Our observations in differentiated PC12 cells suggest that JIP3 associates with a type of anterograde-directed exocytic vesicle at the growth cone that is distinct from neurotransmitter containing synaptic vesicles. We saw no evidence in differentiating PC12 cells for JIP3 association with a retrograde-directed endosomal vesicle or organelle suggested to be involved in signalling axonal injury in other cell types. JIP3 showed significant co-localisation with both JNK and the JNK substrate, paxillin, at the growth cone suggesting that JIP3 may target JNK to the growth cone to phosphorylate paxillin, thus promoting neurite outgrowth.
