# Correction to: JIP3 localises to exocytic vesicles and focal adhesions in the growth cones of differentiated PC12 cells

**DOI:** 10.1007/s11010-018-3354-4

**Published:** 2018-05-03

**Authors:** Patrick T. Caswell, Martin Dickens

**Affiliations:** 1 Wellcome Trust Centre for Cell-Matrix Research, Faculty of Life Sciences, University of Manchester, Manchester, M13 9PT UK; 2 Human Physiology, College of Health, Massey University, North Shore, Auckland, 0630 New Zealand

## Correction to: Molecular and Cellular Biochemistry 10.1007/s11010-017-3222-7

The article “JIP3 localises to exocytic vesicles and focal adhesions in the growth cones of differentiated PC12 cells”, written by “Patrick T. Caswell, Martin Dickens”, was originally published electronically on the publisher’s internet portal https://link.springer.com/article/10.1007/s11010-017-3222-7 on 20 November 2017 without open access.

With the author(s)’ decision to opt for Open Choice the copyright of the article changed on 3 May 2018 to © The Author(s) 2018 and the article is forthwith distributed under the terms of the Creative Commons Attribution 4.0 International License (http://creativecommons.org/licenses/by/4.0/), which permits use, duplication, adaptation, distribution and reproduction in any medium or format, as long as you give appropriate credit to the original author(s) and the source, provide a link to the Creative Commons license and indicate if changes were made.

The original article has been corrected.

